# Overeating in Restrained and Unrestrained Eaters

**DOI:** 10.3389/fnut.2020.00030

**Published:** 2020-03-19

**Authors:** Janet Polivy, C. Peter Herman

**Affiliations:** Department of Psychology, University of Toronto, Toronto, ON, Canada

**Keywords:** overeating, restrained eaters, unrestrained eaters, norm violation, personal norm, social norm

For more than 4 decades we have examined overeating in restrained eaters/chronic dieters. In the laboratory, we have provoked overeating in these individuals by disrupting their diets by means of forced, diet-breaking preloads and through manipulations of affective state, cognitive distraction, or tempting food cues [e.g., (1); or see (2) or (3) for a review]. Many of these effects have been confirmed in real-world settings [e.g., (4–8)]. In fact, in a large population-based study, restrained eaters report that they engage in overeating more than do unrestrained eaters (9). This has led us to believe that at least something is known about overeating in restrained and unrestrained eaters. For example, we know that restrained eaters are more distressed or uncomfortable when they eat more than other people do or when they exceed social norms of appropriate amounts to eat (10–12). In addition, a series of studies has shown that inducing restrained eaters to eat high calorie, “fattening” foods in the laboratory results in their going on to overeat by consuming greater amounts (i.e., greater either than non-preloaded restrained eaters or than preloaded unrestrained eaters) of any palatable, tempting food they are served next [e.g., (13–16); or see (17) for a review]. Even simply being perceptually exposed to attractive foods seems to cause restrained eaters to increase their intake [e.g., (18, 19)].

## What is “Overeating?”

It seems as if we know a lot about overeating in restrained eaters. But maybe we don't know as much as we think we do. There are a lot of assumptions made in the laboratory and field studies cited above. One assumption is that if people eat more after we manipulate something about the eating situation than they do in the absence of that manipulation, that means that in the condition where they eat more they are “overeating.” But what is overeating? Is overeating eating more than some specific, identifiable amount, or does it vary situationally? One would have to agree that someone who eats a large, fully-dressed burger with fries, salad and a milkshake is eating a lot, but is that overeating? If this is that person's dinner, is it overeating? If it is the only meal that the person eats that day, is it overeating? Probably not in either of those two cases; but if it is a snack between lunch and dinner, we would clearly view it as excessive. Moreover, the timing of the eating matters. If someone eats a steak, a large bowl of pasta (with sauce), 3 eggs, 3 pieces of buttered toast, 3 slices of bacon, a milkshake, a piece of cake, an apple, a banana, and a candy bar, that might be seen as overeating. But if that constituted 3 meals and 3 snacks spread out over the course of a day, it might not be considered overeating. And if that person ate that food and then competed in an Iron Man Triathlon, the activity level might require such a large meal, thus making it appropriate, not overeating. Extending this line of thought even further, the same amount of food may be seen as normal eating or as a binge by the same individual, depending on how the individual is feeling at the time (20). So, overeating is at least in some respects a relative term. The fact that restrained eaters report that they overeat more frequently than do unrestrained eaters (9) may simply mean that restrained eaters are more likely to label a given amount of consumption as overeating than are unrestrained eaters [although the fact that dietary restraint does predict weight gain in some prospective studies argues that at least some of the time they are correct, see e.g., (21)].

Overeating is relative for both restrained and unrestrained eaters. In experimental settings, we say that the restrained eaters have overeaten when they eat more in one condition than they do in another, or when they eat more than unrestrained eaters do in the same condition; but when unrestrained eaters eat more than restrained eaters do (in a control condition, for example), we say that the restrained eaters are undereating, rather than considering the possibility that the unrestrained eaters may themselves be overeating. For example, the original preload studies asked participants to arrive having not eaten for 2 or 3 h, but often this meant coming in between lunch and dinner. Non-preloaded unrestrained eaters tended to eat a fairly large amount of ice cream, at least compared to what non-preloaded restrained eaters consumed, or to what preloaded unrestrained eaters ate. But given that this ice cream was in effect a between-meals snack, can we safely conclude that the non-preloaded unrestrained eaters who ate as much as 250 grams of ice cream were not overeating? Conversely, we say that the restrained eaters who ate more ice cream after a milkshake preload than did those not preloaded were overeating, but as these are dieters who may not have eaten anything yet that day, is this necessarily true?

Thus, it is not always obvious what overeating is, and we (and other researchers) may have been somewhat cavalier with our use of the term. We should thus think more explicitly about what we mean when we say that restrained eaters have overeaten. Do we mean that they have eaten more than they intended to eat or than their diets allow (i.e., they have violated a personal eating norm)? Perhaps they have eaten more than others eating in an identical situation, thus violating a social norm of how much should be consumed. Or possibly they have eaten more than a societal norm dictates. As an example, consider the person who eats 2 restaurant main courses. Is such a person an overeater? Or what about people who eat more than is necessary to sustain oneself or than is physically comfortable? As is evident, there are many ways in which one can overeat, depending on how “appropriate” or “normal” eating (i.e., *not* overeating) is being defined in the particular situation.

It is also important to distinguish between “overeating” and “disinhibited eating.” Anyone can overeat (at least using the definition of eating more than is physically necessary or comfortable), but only someone whose eating is inhibited (usually by a weight-loss diet) can be disinhibited. When restrained eaters are disinhibited, whether by believing their diets have already been violated [e.g., (22)], or by emotional provocation [e.g., (23)], alcohol intake [e.g., (24)], or increased cognitive load [e.g., (25)], they increase their consumption (as long as there is palatable food available). Once again, we must question whether this increased eating constitutes overeating or not. In some instances, the amounts consumed may well be great enough to be seen as excessive, but it is not always obvious that such is the case; in fact, it is seldom obvious. Disinhibition is not necessarily equivalent to overeating except by the definition of violating one's own standards or intentions. Herman et al. (26) pointed out that there are two types of overeating, environmentally induced (by the presence of large quantities of tempting food) and disinhibited (as when restrained eaters violate their diets). We must be careful in our use of terminology.

Vartanian et al. (27) recently investigated how two groups of people (university students and a more diverse community sample) defined “appropriate eating.” Participants were asked to indicate how various statements about eating fit into the category of “appropriate food intake” or “normal food intake.” The consensus for both groups was that “appropriate/normal” eating involved responding to hunger or satiety, or maintaining health through nutritious eating. Appropriate eating, then, was seen as eating that is responsive to the internal needs of the body. External norms, cues, the behavior of others, or other social influences on eating behavior were not rated as being part of “appropriate eating.”

Despite decades of research on the strength of social factors in determining eating behavior [e.g, (10, 28–33)], even when asked specifically about such influences, people seem unaware or unable to acknowledge that eating is not solely, or even primarily, determined by physical factors (34). Does this mean that “appropriate eating” is simply determined by hunger and satiety? Not if we look at how people actually behave around food. People appear to be very careful to match their eating to the amount eaten by others around them (33). For example, correlational studies show that people eating together tend to eat similar amounts [e.g., (26, 35, 36)], and experimental studies have consistently shown that people alter their food intake to eat similarly to a “model” who eats small or large amounts (33, 37). This happens whether the participants are especially hungry (38–40) or not (33, 37). It seems, then, that people decide what an appropriate amount to eat is by monitoring the food intake of the people around them. Thus the “appropriate amount” to eat, and therefore the amount that is by definition not excessive or overeating, may be defined by the behavior of other eaters. In the modeling studies reviewed by Cruwyz et al. (37) and Vartanian et al. (33), people consistently ate smaller amounts when eating with others who ate minimally and greater amounts when eating with those who ate large amounts (although still generally less than the large amount eaten by the model). Herman et al. (41) argue that normal or appropriate eating is often defined in terms of the amount eaten by one's eating companions. In this case, overeating is eating more than one's eating companions do. Overeating, then, may be construed in various ways.

We proposed that there are different ways to overeat, and thus different types of overeating. One can literally eat too much for one's body to comfortably process, which is true physical overeating. But as the research mentioned above suggests, there are other forms of overeating as well. When restrained eaters or dieters violate their diet goals or personal conceptions of what is enough or appropriate to eat, they may feel that they have overeaten, even if the actual quantity eaten is less than would be eaten by someone without dietary restrictions (26). If asked, such dieters would most likely call their consumption overeating. Finally, as the modeling studies above demonstrate, the amount eaten by others can define a norm, and violating this socially defined norm can be seen by the self and others as overeating, again, even if the quantities involved are small and less than required for the individual's own satiety (41). There are thus at least three kinds of overeating. We will look at these more closely below.

## Types of Overeating

### Eating More Than Is Physically Needed/Desirable

We all know the feeling of having eaten too much, to the point that we feel uncomfortable. Just think of your last holiday dinner with friends or family. Children let loose on large amounts of candy or other preferred foods may overeat until they literally reach the limits of their physical capacity and vomit. Most of the time, however, we (try to) gauge our portion sizes to meet our fullness goals, and we use our expectations of how filling the food available will be to decide how much to eat (42). But there are many circumstances that can interfere with our plans and lead us to eat more. As mentioned, holiday celebrations tend to result in overeating and even weight gain (43–46). Even being distracted while eating can result in increased intake, especially among restrained eaters, who may not even be aware that they have eaten more than usual [e.g., (25, 47–50)]. (It's not clear whether being distracted prevents the eater from accurately monitoring (a) how much food she is eating or (b) accumulating satiety cues. In either case, the result is excessive intake.) Being confronted with large portions also reliably induces people to increase their consumption (again, often without their awareness) until they are uncomfortably full [e.g., (51–54)]. Thus, overeating to the point of over-satiation can occur without conscious intent or awareness, depending on the eating situation. Still, this is a matter of true physical overeating.

When we speak of overeating, we are generally talking about a single eating episode or meal. But if one overeats at one meal and then compensates for the rest of the day by undereating, thus achieving a total caloric intake for the day that matches that of people who ate moderate amounts at all meals, did the person overeat? They may have felt uncomfortably full at the time of the “overeating episode” which caused them to undereat for the rest of the day, thus arriving at a “normal” intake for that day, or they may have decided to compensate for other reasons, but the discomfort of overeating at one point in the day did nonetheless occur. Does compensatory undereating for the rest of the day, and thus keeping the total intake from being excessive, or more than the person needed, essentially mean that the overeating that did occur does not have any further implications (and thus it is as if it never did occur)? In such an instance, did overeating occur or not? According to Woods (39), even when one compensates by eating less over the rest of the day, an episode of overeating does have effects on the body. The body learns to anticipate the arrival of food into the body from the presence of food cues. If one eats more or less than one usually does, the body does not secrete the appropriate amount of insulin, as the insulin secretion response has been learned over time, so that the insulin is sufficient to prevent too much glucose from entering the bloodstream. When one “overeats,” even just at a single meal, excess glucose enters the bloodstream, rendering the person essentially diabetic for as long as it takes the body to manufacture and release more insulin to clear out the excess glucose. Woods notes that while there is no clear reason why elevated glucose should be dangerous to animals (and people), the tight physiological regulation of consumption-produced glucose indicates that such elevations must pose a risk to health. Thus, even when one compensates for having overeaten, there may still be negative effects and thus it seems that overeating at a single occasion is still, physically speaking, overeating, even if the consumption for the rest of the day is curtailed.

Is physical overeating defined by the same quantities for all individuals? When asked what the appropriate amount to eat should be, both university students and a community sample of adults agreed that the nutritional needs of the individual determine what is appropriate (27). Obviously, athletes on training regimes who are expending huge numbers of calories need to eat more than office workers who sit at a desk all day. Moreover, individual differences in body size, composition, and metabolic rate, as well as exercise or caloric expenditure level, will determine how many calories a given individual needs simply to maintain caloric equilibrium. So physical overeating cannot be defined as any particular amount of food, as the amounts that constitute appropriate and excessive intake will differ from individual to individual.

What are the effects of such true overeating? Not surprisingly, people who overeat, by eating more than is needed or wanted by their bodies, feel physically uncomfortable and eventually stop eating, or may even vomit if their overconsumption pushed them beyond what their stomachs could hold. This is true for both restrained and unrestrained eaters. When given a large milkshake preload (15 ounces of milkshake), unrestrained eaters reduced their intake, but restrained eaters ate more of the subsequently presented food that they were asked to “taste and rate” (13, 22). After a doubly large preload (30 ounces of milkshake), however, both restrained and unrestrained eaters ate minimal amounts of the taste-rating food (13). Thus, overeating in restrained eaters does not inevitably result in more overeating, especially if the initial overeating is severe enough to produce physical satiety. Over the long term, it is not clear whether dieters are more likely to overeat physically than are non-dieters and thus weigh more, or if they are merely genetically more inclined to maintain or gain weight on smaller quantities of food (26).

### Personal Norm Violation

When asked if they have overeaten or how often they overeat, most people (or at least most dieters) are probably thinking about eating more than they intended or expected to eat, not necessarily eating to the point of physical discomfort (29). People differ in their satiety goals when they eat. In some instances, they simply wish to forestall hunger; but in general, people want to feel comfortably full or sated after they eat, although there are some people who want to feel completely full (55). Although these individuals may eat different amounts, none of them could be said to have overeaten with respect to a personal eating goal.

Dieters, or restrained eaters, however, set themselves goals that are designed to help them to lose weight or maintain a lowered weight, and such eating goals often involve eating less food than they would like (56). Decades of studies of eating in dieters document how dieters can be induced to succumb to temptation and eat more than they planned. Not only do dieters eat more when they have been induced to consume a diet-breaking preload, but their diets are disrupted by emotions [e.g., (1, 57, 58)], alcohol (24, 59), or cognitive load/distraction (60); even merely seeing (19, 61, 62) or smelling (63) tempting foods can make restrained eaters abandon their diets. These disruptions of dietary restraint lead restrained eaters to abandon their diet goals for at least the short term, and instead to indulge in the foods they have been denying themselves. Herman and Polivy (56) called this the “what the hell effect.” They posited that it is as if dieters are saying to themselves something to the effect of, “what the hell, my diet is already broken so I might as well eat and enjoy for now.” Stroebe et al. (64, 65) have argued that another possibility is that restrained eaters actually have 2 conflicting goals, a dieting goal and an eating goal; when the dieting goal is violated or abandoned, they are free to pursue the eating goal (Stroebe et al.'s interpretation is by no means incompatible with Herman and Polivy's interpretation). The question, then, is whether the eating goal produces real overeating when dieting restraints are loosened. It may be that restrained eaters don't actually overeat more than unrestrained eaters do but rather are more likely to consider a given amount eaten to be “overeating.”

For some individuals, particularly for restrained eaters, overeating may mean simply eating any amount of a food that is seen as unacceptable or “forbidden” by one's dietary regimen (15). Knight and Boland found that restrained eaters rated significantly more foods as forbidden than did unrestrained eaters. Moreover, when presented with preloads of equivalent caloric value, restrained eaters went on to eat more (i.e. “overeat”) only if the preload foods were forbidden foods, regardless of their caloric content, whereas unrestrained eaters responded simply to the calorie content of the foods. Further, restrained (but not unrestrained) eaters increased their intake when they merely *anticipated* having to eat some of a forbidden food, again, regardless of caloric content. Thus, any amount of a forbidden food may be too much for one's diet to tolerate, and thus represents overeating for a chronic dieter. In fact, most laboratory studies of overeating in restrained eaters deliberately use foods seen by most people as inherently fattening or diet-breaking, irrespective of amount [e.g., (2, 25, 56, 66)].

When we do eat more than we planned to eat, or indulge in a tempting food we consider to be forbidden, there are several possible outcomes we may experience. Such eating could make us feel physically uncomfortable (i.e., “stuffed”) when we truly eat larger amounts of food (as described in the previous section). However, if the overconsumption represents violating a restrictive diet or other eating goal rather than eating an objectively excessive amount of food, we may feel uncomfortable psychologically, guilty and disappointed in ourselves for not exhibiting self-control, despite not having eaten an objectively large amount of food [e.g., (3, 67, 68)]. The guilt or dysphoria produced by the initial transgression may then lead to continued eating or even objective overeating; once they think that they have already “overeaten” or transgressed their diet goal, restrained eaters are likely to continue eating [(3, 17, 69)].

### Social Norm Violation: Eating More Than Other Eaters

When we eat with others (as people do most of the time), our eating is very much guided by what and how much those others eat (29). For the most part, we seem to try to limit our intake so that we do not exceed the amount eaten by others (10). In fact, the large literature on modeling of eating behavior shows that people very consistently eat (somewhat) less than those with whom they are eating, especially when eating with confederates who have been instructed to eat a lot (29).

Violating social norms by eating more than one's eating companions do seems to be something people work hard to avoid. We may be worried that we will be viewed as large eaters, a label that is associated with being regarded as unattractive, lacking in self-control, and unfeminine compared to those viewed as small eaters (70). In addition, young people who report that they tend to overeat relative to others also report that they generally use less healthy dieting methods, and feel worse about themselves, being more depressed and dissatisfied with themselves and their bodies, as well as having lower self-esteem (71). At the same time, however, we tend to like other people who eat more than we do more, and dislike those who eat less than we do (72); if someone eats more than we do, then by comparison we become reasonable eaters, whereas if someone eats less than we do, it makes us look bad. Moreover, if we are given a smaller or larger portion than others eating with us are given, this affects how we feel about our eating and how we behave subsequently (12). Restrained eaters who get what appears to be a larger portion eat more of a subsequent food than those getting a smaller or similar portion (and than unrestrained eaters who get the larger portion), although they did not feel badly about themselves and were actually happier than those who got what they thought was a smaller portion. Thus, overeating relative to others may result in social rejection or embarrassment, although some people may secretly like us for allowing them to eat more or feel good when someone else “forces” them to eat more. But this does not seem to be enough to counteract the fear of being seen as an overeater. An experimental investigation of people's reasons for controlling their consumption showed that it was those who were thinking about avoiding negative outcomes who most closely matched their eating to other eaters in the situation (73).

For restrained eaters who are particularly responsive to food cues (19, 62, 74) and who tend to like fattening foods more (75), it may be particularly difficult to avoid succumbing to the temptation to eat more of tempting, attractive foods. The social sanctions seem to be strong enough to prevent overeating when being watched by others (76, 77), when one has to report to others how much one ate (78), or even when just eating in front of a statue of a human head (79). But restrained eaters eating on their own are less able to resist tempting foods (or emotional upsets, or distractions or other disruptions of their self-control), and are prone to eating more, possibly to the extent of actual overeating [e.g., (66)].

## What do we Know About Overeating?

It seems that researchers (including ourselves) have been conflating the various types of overeating in studies and discussions of the concept. What does this mean for our understanding and even recognition of overeating? Clearly, we need to be more specific in our definitions of what we are investigating and what we are trying to describe. Physical or objective overeating, self-perceived overeating, and simply eating more than others around you are arguably three different phenomena. The outcomes of the three types of overeating differ in some ways. Physically overeating leads to physical discomfort, which is not generally the case for those who violate a personal or social eating norm. Violating a personal norm leads restrained eaters to continue eating, possibly because it gives them an excuse to go ahead and eat the foods that they normally deny themselves. Violation of a social norm seems, on the other hand, to lead to less eating, in order to avoid seeming greedy or piggish. But violating a norm, either social or personal, seems to produce similar feelings of guilt, embarrassment, and disappointment in oneself. One could thus say that despite the differences in quality of discomfort (psychological vs. physical), all overeating does seem to exact a price.

## Author Contributions

JP and CH discussed the content of the paper. JP wrote the first draft and CH commented, corrected, and made suggestions, which JP then incorporated into the manuscript. JP thus did 75% of the work and CH did 25%, which is why JP is first author.

### Conflict of Interest

The authors declare that the research was conducted in the absence of any commercial or financial relationships that could be construed as a potential conflict of interest. The reviewer TV declared a past co-authorship with the authors to the handling Editor.
